# Diffusion MRI Is More Sensitive Than Tau PET and Regional Volume Loss in Corticobasal Syndrome

**DOI:** 10.64898/2026.07.24.26358494

**Published:** 2026-07-27

**Authors:** Yuqi Tian, Ryota Satoh, Val J. Lowe, Keith A. Josephs, Jennifer L. Whitwell

## Abstract

**Background:**

Corticobasal syndrome (CBS) is a neurodegenerative syndrome that often results from a 4-repeat tauopathy. Abnormalities on tau PET, structural MRI, and diffusion MRI (dMRI) have been observed in CBS, although no prior work has systematically compared the relative sensitivity of these modalities. In addition, the relationships between tau uptake, volume loss, and white matter degeneration remain incompletely understood.

**Objective:**

To compare the sensitivity of tau PET, structural volume, and dMRI metrics to differentiate CBS from controls, and to characterize relationships between regional tau uptake, volume loss, and white matter tract abnormalities.

**Methods:**

Thirty-two participants meeting criteria for possible or probable CBS and 35 healthy controls underwent standardized 3T dMRI, ^18^ F-flortaucipir tau PET, and detailed neurologic assessment. Diffusion data were processed using complementary diffusion tensor, free-water, and Neurite Orientation Dispersion and Density Imaging (NODDI) pipelines, with tract-level metrics extracted using the Johns Hopkins University-Eve (JHU EVE) White Matter atlas. Regional gray matter volumes and flortaucipir standardized uptake value ratios (SUVRs) were calculated. Global tau PET uptake, represented by the first principal component (PC1), was removed in a secondary tau analysis. Modality-level discrimination was assessed using area under the curve (AUC) analyses. Cross-modality relationships were evaluated using regional correlations and covariate-adjusted models comparing volume- and tau-related predictors of white matter abnormalities.

**Results:**

Relative to controls, CBS showed widespread higher mean diffusivity (MD) and lower intracellular volume fraction (ICVF), especially in sensorimotor and projection white matter pathways. Structural volume reductions were most prominent in precentral cortex and subcortical regions including putamen, thalamus, and pallidum, whereas tau PET abnormalities were weaker and less spatially extensive. MD showed the strongest overall discrimination between CBS and controls, followed by ICVF, structural volume, PC1-removed tau PET, and original tau PET. In targeted sensorimotor analyses, higher DTI-MD was most strongly associated with lower gray matter volume. PC1-removed sensorimotor tau uptake was also associated with higher sensorimotor DTI-MD, whereas original tau uptake and broader cortical or subcortical tau measures were not significant predictors.

**Conclusions:**

White matter microstructural disruption is a dominant imaging signature of CBS and is more closely linked to gray matter volume loss than to measurable uptake on tau PET. dMRI, particularly MD and ICVF, may provide greatest sensitivity for detecting disease-related changes in CBS.

## Full Text Availability

The license terms selected by the author(s) for this preprint version do not permit archiving in PMC. The full text is available from the preprint server.

